# Infectious Etiologies and Antimicrobial Management of Acute Chest Syndrome in Adult Sickle Cell Disease Patients: Pathogen Identification Patterns and Clinical Outcomes from a Five-Year Retrospective Study in Eastern Saudi Arabia

**DOI:** 10.3390/pathogens14111174

**Published:** 2025-11-18

**Authors:** Ali Alsaeed, Reda Aleid, Omar Amin, Amjad Alansari, Hadi Aleid, Mohammed Aleid

**Affiliations:** 1 Infectious Disease Section, Internal Medicine Department, Dammam Medical Complex, Dammam 32241, Saudi Arabia; 2 Hematology Section, Internal Medicine Department, Dammam Medical Complex, Dammam 32241, Saudi Arabia; 3 Internal Medicine Department, Dammam Medical Complex, Dammam 32241, Saudi Arabia; 4 Data Analysis Unit, Dammam Medical Complex, Dammam 32241, Saudi Arabia; 5 Department of Medicine, Royal College of Surgeons in Ireland, D02 YN77 Dublin, Ireland; 6 Intensive Care Unit, Dammam Medical Complex, Dammam 32241, Saudi Arabia

**Keywords:** acute chest syndrome, antimicrobial therapy, bacterial pathogens, immunocompromised host, infectious complications, Mycoplasma pneumoniae, sickle cell disease, vaccination

## Abstract

Acute chest syndrome (ACS) is a life-threatening complication of sickle cell disease (SCD) with complex infectious and non-infectious etiologies. Bacterial pathogens, including Streptococcus pneumoniae, Haemophilus influenzae, and atypical organisms such as Mycoplasma pneumoniae, play crucial roles in ACS pathogenesis, particularly in immunocompromised SCD patients with functional asplenia. Despite the importance of infectious triggers, regional data on pathogen identification rates and antimicrobial management strategies in ACS remain limited, especially from high-prevalence SCD regions. This study aimed to investigate the infectious etiologies, pathogen identification patterns, and antimicrobial management outcomes of ACS in adult SCD patients in Eastern Saudi Arabia. A five-year retrospective analysis was conducted on patients aged ≥14 years with SCD who were admitted with ACS to Dammam Medical Complex between 2018 and 2022. Comprehensive microbiological evaluation included blood cultures, sputum cultures, and atypical pathogen testing (Mycoplasma pneumoniae, Chlamydia pneumoniae). Data on antimicrobial regimens, pathogen identification rates, vaccination status against encapsulated bacteria, and clinical outcomes were systematically analyzed. Empirical antibiotic strategies and their effectiveness in this immunocompromised population were evaluated. A total of 60 adult SCD patients experiencing 80 episodes of ACS were included. Despite comprehensive microbiological workup, specific infectious pathogens were identified in only 8 (10.0%) episodes, highlighting the complex multifactorial etiology of ACS. Blood cultures yielded pathogens in 5 (6.3%) cases, sputum cultures in 4 (5.0%) cases, and Mycoplasma pneumoniae was identified in 3 (3.8%) episodes. All patients received empirical broad-spectrum antimicrobial therapy, with ceftriaxone and azithromycin combination being the most frequent regimen (76 cases, 95.0%), providing coverage for both typical and atypical bacterial pathogens. Antibiotic escalation was required in 16 (20.0%) episodes. Vaccination rates against Streptococcus pneumoniae were suboptimal at 30 (50.0%), representing a significant risk factor for invasive bacterial infections in this functionally asplenic population. The intensive care unit (ICU) admission rate was 15 (18.8%), and in-hospital mortality was 3 (3.8%), with infectious complications contributing to severe outcomes. In this cohort of SCD patients, ACS demonstrated low rates of specific pathogen identification despite systematic microbiological investigation, supporting the multifactorial infectious and non-infectious etiology of this syndrome. The predominant use of broad-spectrum antimicrobial therapy targeting both typical and atypical bacterial pathogens proved effective in this immunocompromised population. However, suboptimal vaccination rates against encapsulated bacteria represent a critical gap in infection prevention strategies. These findings emphasize the importance of empirical antimicrobial coverage for suspected bacterial pathogens in ACS management and highlight the urgent need for enhanced vaccination programs to prevent infectious complications in functionally asplenic SCD patients.

## 1. Introduction

Acute chest syndrome (ACS) is a life-threatening complication of sickle cell disease (SCD) and a leading cause of hospitalization and mortality in affected individuals [1]. It is defined by the presence of a new pulmonary infiltrate on chest imaging, accompanied by fever and/or respiratory symptoms such as chest pain, cough, or dyspnea [1]. The clinical presentation of ACS can be severe and may progress rapidly to acute respiratory failure, often necessitating intensive care unit (ICU) admission and mechanical ventilation [2]. Previous studies have reported mortality rates as high as 3% in the general ACS population, with adults having a more severe disease course and worse outcomes compared to children [2].

The pathophysiology of ACS is complex and multifactorial, involving a combination of infection, fat embolism from bone marrow infarction, and pulmonary vaso-occlusion due to sickled erythrocytes [1]. Identifying a specific etiology for each ACS episode can be challenging, and in many cases, a definitive cause is not found [2]. Atypical bacteria such as Mycoplasma pneumoniae and Chlamydia pneumoniae, as well as respiratory viruses, are frequently implicated, while typical bacteria like Streptococcus pneumoniae and Haemophilus influenzae are also recognized pathogens, particularly in asplenic patients [3,4].

Beyond the acute complications of ACS, patients living with SCD experience a substantial burden of chronic complications that significantly impact their quality of life and long-term outcomes. These include cardiovascular disease, with an increased risk of pulmonary hypertension and cardiomyopathy; thromboembolic events due to the hypercoagulable state associated with chronic hemolysis; endocrinopathies such as hypothyroidism and hypogonadism; and complications related to chronic transfusion therapy, including iron overload and alloimmunization [5,6]. The cumulative effect of these complications, combined with the infectious susceptibility inherent to functional asplenia, creates a complex clinical picture that requires comprehensive multidisciplinary management.

The infectious susceptibility of SCD patients is significantly increased due to functional asplenia, which develops early in life as a result of repeated splenic infarctions [5]. This immunocompromised state predisposes patients to severe infections with encapsulated bacteria, making vaccination against Streptococcus pneumoniae, Haemophilus influenzae, and Neisseria meningitidis crucial for infection prevention [6]. The interplay between infectious triggers and the underlying pathophysiology of SCD creates a complex clinical scenario where empirical antimicrobial therapy must balance broad-spectrum coverage with antimicrobial stewardship principles [7].

Geographic and genetic factors can influence the clinical manifestations and outcomes of SCD and its complications. The Eastern Province of Saudi Arabia has one of the highest rates of SCD in the country, with a prevalence of up to 2.6% in some areas [8]. The SCD in this region is predominantly of the Arab-Indian haplotype, which is often associated with higher levels of fetal hemoglobin and a milder clinical phenotype compared to the African haplotypes [9]. While earlier research from this region suggested a lower frequency and severity of ACS, it remains a significant cause of morbidity and mortality [9,10]. However, there is a lack of recent data on adult ACS from Eastern Saudi Arabia, with most studies being either outdated or focused on pediatric populations [11,12].

Given the potential for regional variations in the presentation and outcomes of ACS, and the improvements in SCD care over the last two decades, there is a critical knowledge gap regarding contemporary adult ACS data from high-prevalence regions. Most existing studies from Eastern Saudi Arabia are either outdated or focus on pediatric populations, leaving a significant void in our understanding of current infectious etiologies, antimicrobial resistance patterns, and clinical outcomes in adult patients. This study addresses this gap by providing the first comprehensive analysis of ACS in adult SCD patients from this region in over a decade, offering valuable insights into current pathogen identification patterns and antimicrobial management strategies in the modern era of SCD care. This study aims to describe the infectious etiologies, pathogen identification patterns, antimicrobial management strategies, and clinical outcomes of adult patients with ACS in a tertiary care center in Eastern Saudi Arabia, with the goal of providing valuable regional data to inform clinical practice and identify areas for improvement in infection prevention and antimicrobial management.

## 2. Materials and Methods

### 2.1. Study Design and Population

We conducted a retrospective cohort study of all patients aged ≥14 years with a diagnosis of SCD who were admitted with ACS to Dammam Medical Complex, a tertiary care center in Eastern Saudi Arabia, between 1 January 2018 and 31 December 2022. Patients were identified through a search of the hospital’s electronic medical records using the International Classification of Diseases, Tenth Revision (ICD-10) discharge codes for ACS (D57.0x) [13]. The diagnosis of ACS was then manually verified for each patient by reviewing their medical chart for the presence of a new pulmonary infiltrate on chest radiography, accompanied by fever (temperature > 38.3 °C) and/or respiratory symptoms (chest pain, cough, or dyspnea), consistent with established diagnostic criteria [1,2]. All patients were admitted under the care of the general hospitalist (internal medicine) service with infectious disease consultation when indicated.

### 2.2. Microbiological Evaluation

Comprehensive microbiological workup was performed for all patients according to institutional protocols. Blood cultures were obtained from all patients upon admission, with at least two sets (aerobic and anaerobic bottles) collected from separate venipuncture sites to minimize contamination risk, with additional cultures drawn if clinically indicated. Potential culture contamination was ruled out based on established criteria, including assessment of organism type, number of positive culture sets, time to positivity, and clinical correlation. Sputum samples were collected when feasible and processed for bacterial culture and sensitivity testing. Testing for atypical pathogens, including Mycoplasma pneumoniae and Chlamydia pneumoniae, was performed using polymerase chain reaction (PCR) or serological methods when clinically suspected. Viral respiratory panel testing was conducted selectively based on clinical presentation and seasonal patterns.

### 2.3. Data Collection

Data were extracted from the electronic medical records and entered into a standardized data collection form. The collected data included baseline characteristics such as age, sex, SCD genotype, and use of hydroxyurea at baseline. Vaccination status was documented for Streptococcus pneumoniae, influenza, and COVID-19. Clinical presentation data included whether ACS was present on admission or developed during hospitalization, association with a vaso-occlusive crisis, presenting symptoms, and vital signs. Laboratory findings encompassed complete blood count with differential and arterial blood gas analysis. Radiological findings included the location and characteristics of pulmonary infiltrates on chest X-ray and presence of pleural effusion. Management details covered antibiotic therapy, including initial empiric regimens and any subsequent escalations, use of supplemental oxygen, non-invasive ventilation, blood transfusions, and other supportive measures. Microbiological data included results from blood and sputum cultures, as well as testing for atypical pathogens. Outcomes measured included requirement for ICU admission, need for mechanical ventilation, length of hospital stay, in-hospital mortality, and 30-day readmission rates.

### 2.4. Statistical Analysis

Descriptive statistics were used to summarize the patient characteristics, clinical features, management strategies, and outcomes [14]. The normality of distribution for continuous variables was assessed using the Shapiro–Wilk test and visual inspection of histograms and Q-Q plots. Normally distributed continuous variables are presented as means and standard deviations (SD), while non-normally distributed variables are presented as medians and interquartile ranges (IQR). Categorical variables are presented as frequencies and percentages. Missing data were handled using complete case analysis, with the proportion of missing data reported for each variable when applicable. Comparisons between subgroups (e.g., patients who required ICU admission versus those who did not) were performed using the chi-square test or Fisher’s exact test for categorical variables and the t-test or Mann–Whitney U test for continuous variables, as appropriate [15]. A *p*-value of <0.05 was considered statistically significant. Antibiotic escalation was defined as a change from the initial empirical regimen to broader-spectrum agents or addition of antimicrobial coverage based on clinical deterioration, microbiological findings, or infectious disease consultation recommendations. All statistical analyses were performed using SPSS version 26 (IBM Corp., Armonk, NY, USA), which was selected for its robust statistical capabilities and widespread use in clinical research.

### 2.5. Ethical Considerations

This study was conducted in accordance with the Declaration of Helsinki and was approved by the Institutional Review Board of Dammam Medical Complex. Given the retrospective nature of the study, informed consent was waived. Patient confidentiality was maintained throughout the study, and all data were de-identified prior to analysis.

## 3. Results

### 3.1. Patient Characteristics and Baseline Infectious Risk Factors

A total of 60 SCD patients aged ≥14 years who experienced 80 episodes of ACS were included in the study (Table 1). Among these patients, 40 (66.7%) experienced a single ACS episode during the study period, 14 (23.3%) experienced two episodes, 4 (6.7%) experienced three episodes, and 2 (3.3%) experienced four or more episodes. The mean age of the patients was 24.6 ± 8.3 years, and 35 (58.3%) were male. The majority of patients (52, 86.7%) had homozygous SCD (HbSS), while the remaining 8 (13.3%) had other genotypes such as HbS-β-thalassemia. At baseline, only 20 (33.3%) patients were on hydroxyurea therapy. Vaccination coverage against encapsulated bacteria was found to be suboptimal in this immunocompromised cohort. Only 30 (50.0%) patients had received a pneumococcal vaccine, representing a significant gap in protection against Streptococcus pneumoniae infections. Additionally, 24 (40.0%) had received at least one dose of a COVID-19 vaccine, and only 18 (30.0%) had documented evidence of annual influenza vaccination.

### 3.2. Clinical Presentation and Infectious Manifestations

ACS was the reason for admission in 60 (75.0%) of the episodes, while in the remaining 20 (25.0%) episodes, it developed during hospitalization for other reasons (Table 2). A concurrent vaso-occlusive crisis (VOC) was present in 72 (90.0%) of all ACS episodes, suggesting a strong association between pain crises and the development of ACS. The most frequently reported presenting symptoms were chest pain 64 (80.0%), shortness of breath 60 (75.0%), fever 48 (60.0%), and cough 44 (55.0%). The high prevalence of fever (60%) suggests a significant inflammatory or infectious component in the majority of cases. On physical examination, tachypnea was observed in 56 (70.0%) episodes, and 40 (50.0%) patients had an oxygen saturation of less than 92% on room air, indicating significant respiratory compromise. The most common radiological finding was a unilateral pulmonary infiltrate, present in 56 (70.0%) cases, while 24 (30.0%) had bilateral infiltrates. Pleural effusions were noted in 16 (20.0%) cases.

Laboratory investigations revealed a significant drop in hemoglobin levels during ACS episodes compared to the patients’ baseline values (mean 7.9 ± 1.2 g/dL vs. 9.1 ± 1.3 g/dL; *p* < 0.01). Leukocytosis was a common finding, with a mean white blood cell count of 15.2 × 10^9^/L, reflecting the inflammatory response characteristic of ACS. Thrombocytosis was observed in 52 (65.0%) of the episodes, with a mean platelet count of 425 ± 145 × 10^9^/L (range: 150–850 × 10^9^/L). Arterial blood gas analysis showed hypoxemia (PaO_2_ < 70 mmHg) in 24 (30.0%) cases.

### 3.3. Microbiological Findings and Pathogen Identification

Despite comprehensive microbiological evaluation, specific infectious pathogens were identified in only 8 (10.0%) of the 80 ACS episodes (Table 3). Blood cultures were positive in 5 (6.3%) cases, yielding organisms including Streptococcus pneumoniae (2 cases), Staphylococcus aureus (2 cases), and Escherichia coli (1 case). Sputum cultures yielded a pathogen in 4 (5.0%) cases, with similar organisms identified. Mycoplasma pneumoniae was specifically identified in 3 (3.8%) cases through PCR testing, confirming its role as an important atypical pathogen in this population. The low overall pathogen identification rate (10.0%) highlights the complex multifactorial etiology of ACS, where infectious triggers may be present but difficult to detect, or where non-infectious mechanisms such as fat embolism and vaso-occlusion predominate. The identification of Mycoplasma pneumoniae in 3.8% of cases is consistent with its recognized role as an atypical pathogen in ACS, particularly important given the need for specific antimicrobial coverage.

### 3.4. Antimicrobial Management Strategies

All 80 ACS episodes were treated with empirical broad-spectrum antimicrobial therapy (Table 4). The most common initial antibiotic regimen was a combination of ceftriaxone and azithromycin, used in 76 (95.0%) episodes. This regimen provides comprehensive coverage for typical bacterial pathogens (Streptococcus pneumoniae, Haemophilus influenzae) as well as atypical organisms (Mycoplasma pneumoniae, Chlamydia pneumoniae), making it an appropriate empirical choice for this immunocompromised population. In 16 (20.0%) episodes, antibiotic therapy was escalated to broader-spectrum agents, typically due to clinical deterioration or concern for resistant organisms. Infectious disease consultation was obtained in these cases to guide appropriate antimicrobial selection. The escalation rate of 20% suggests that while the initial empirical regimen was appropriate for most cases, a significant minority required more intensive antimicrobial management.

### 3.5. Clinical Outcomes and Infectious Complications

The clinical outcomes demonstrated the effectiveness of standardized antimicrobial management protocols in this immunocompromised population. Fifteen (18.8%) of the ACS episodes required admission to the ICU, and 8 (10.0%) patients required mechanical ventilation. The median length of hospital stay was 7 days (interquartile range: 5–11 days). Three (3.8%) deaths occurred during the study period, with infectious complications contributing to the fatal outcomes in 2 of these cases. Blood transfusions were administered in 40 (50.0%) of the ACS episodes, with simple transfusions performed in 32 (40.0%) episodes and exchange transfusions reserved for 8 (10.0%) episodes. All patients received aggressive pain management and incentive spirometry as part of standard care. Bronchodilators were administered in 20 (25.0%) episodes, and supplemental oxygen was required in 44 (55.0%) episodes. Recurrent ACS was observed in 12 (20.0%) patients within the follow-up period, and the 30-day readmission rate was 12 (15.0%). These rates highlight the chronic nature of SCD and the ongoing risk of infectious and non-infectious complications in this population.

### 3.6. Risk Factors for Severe Outcomes

Patients who required ICU admission had significantly different clinical and laboratory parameters compared to those managed on the general medical ward (Table 5). ICU patients had significantly higher admission white blood cell counts (18.3 ± 5.1 × 10^9^/L vs. 14.2 ± 3.9 × 10^9^/L, *p* = 0.004) and lower hemoglobin levels (7.2 ± 1.1 g/dL vs. 8.1 ± 1.2 g/dL, *p* = 0.02). Platelet counts were also significantly different between groups, with ICU patients showing higher thrombocytosis (mean 485 ± 162 × 10^9^/L vs. 405 ± 138 × 10^9^/L, *p* = 0.03). Additionally, ICU patients had lower oxygen saturation on admission (mean 88.2 ± 4.5% vs. 92.5 ± 3.2%, *p* = 0.001) and higher C-reactive protein levels (mean 145 ± 62 mg/L vs. 98 ± 45 mg/L, *p* = 0.008). These findings suggest that more severe inflammatory responses, greater degrees of anemia, thrombocytosis, and hypoxemia may serve as early predictors of disease severity and the need for intensive care management.

## 4. Discussion

### 4.1. Infectious Etiologies and Pathogen Identification Challenges

In this five-year retrospective study of adult SCD patients with ACS in Eastern Saudi Arabia, our findings align with previous reports that demonstrate the complex infectious and non-infectious etiologies of this syndrome [1,2]. The low rate of specific pathogen identification (10.0%) despite comprehensive microbiological workup highlights the multifactorial pathophysiology of ACS, where infectious triggers may be present but difficult to detect using conventional diagnostic methods [16]. This finding is consistent with large multicenter studies that have reported pathogen identification rates ranging from 29% to 38% when comprehensive testing is performed [3,17]. The lower pathogen detection rate in our study compared to these multicenter investigations may be attributed to several factors, including limitations in the sensitivity of conventional microbiological testing methods, the timing of sample collection relative to symptom onset, prior antibiotic exposure in some patients, and the substantial contribution of non-infectious triggers such as fat embolism and pulmonary vaso-occlusion to ACS pathogenesis. Additionally, our selective use of viral respiratory panel testing, rather than universal viral screening, may have resulted in underdetection of viral etiologies that can serve as important triggers for ACS. These findings underscore the need for more sensitive diagnostic approaches, including molecular methods and comprehensive viral testing, to better characterize the infectious burden in ACS.

The identification of Mycoplasma pneumoniae in 3.8% of cases confirms its importance as an atypical pathogen in ACS, particularly relevant given that this organism requires specific antimicrobial coverage that differs from standard anti-pneumococcal therapy [4,18]. The detection of Streptococcus pneumoniae in blood cultures from 2 patients underscores the continued importance of this encapsulated bacterium as a significant pathogen in functionally asplenic SCD patients [19]. The presence of Staphylococcus aureus bacteremia in 2 cases may reflect either primary bloodstream infection or secondary seeding from other infectious foci, highlighting the complex infectious susceptibility of this immunocompromised population [20].

### 4.2. Antimicrobial Management in Immunocompromised SCD Patients

The management of ACS in our cohort followed evidence-based guidelines for antimicrobial therapy in immunocompromised patients, with the vast majority receiving a combination of a third-generation cephalosporin and a macrolide [21,22]. This regimen provides broad-spectrum coverage for typical bacterial pathogens (Streptococcus pneumoniae, Haemophilus influenzae) while also targeting atypical organisms (Mycoplasma pneumoniae, Chlamydia pneumoniae) that are frequently implicated in ACS [23,24].

The antibiotic escalation rate of 20% suggests that while empirical therapy was appropriate for most patients, a significant minority required broader antimicrobial coverage. In cases requiring escalation, infectious disease consultation was obtained to guide appropriate therapy selection, reflecting best practices in antimicrobial stewardship [25]. This approach balances the need for adequate pathogen coverage with the principles of judicious antibiotic use, particularly important in an era of increasing antimicrobial resistance [26].

The use of supportive care measures such as supplemental oxygen, bronchodilators for patients with reactive airway disease, and transfusions for severe cases is consistent with current recommendations for ACS management [2,27,28]. The implementation of incentive spirometry in all patients reflects evidence-based approaches to preventing acute pulmonary complications in SCD [29].

### 4.3. Vaccination Gaps and Infection Prevention Opportunities

A particularly concerning finding from our study is the suboptimal vaccination rates against encapsulated bacteria in this high-risk immunocompromised population. Only 50% of patients had received pneumococcal vaccination, representing a critical gap in infection prevention strategies. Given the functional asplenia that develops in SCD patients, vaccination against Streptococcus pneumoniae, Haemophilus influenzae type b, and Neisseria meningitidis is considered a cornerstone of preventive care [30,31].

The low influenza vaccination rate (30%) is equally concerning, as respiratory viral infections can serve as triggers for ACS and predispose to secondary bacterial infections [32]. The COVID-19 vaccination rate of 40% reflects the timing of our study period and the evolving availability of vaccines, but emphasizes the importance of comprehensive vaccination strategies in this vulnerable population [33].

Improving vaccination coverage should be a key public health priority in this region, as studies have demonstrated significant reductions in invasive bacterial infections following implementation of comprehensive vaccination programs in SCD patients [34]. The establishment of systematic vaccination protocols and patient education programs could substantially reduce the incidence of infectious complications in this population [35]. The findings from this study have direct implications for antimicrobial stewardship and vaccination policy improvements in the region. Specifically, the establishment of standardized empirical antimicrobial protocols that balance broad-spectrum coverage with stewardship principles can optimize outcomes while minimizing resistance development. Furthermore, the implementation of systematic vaccination programs, including pneumococcal vaccination for all SCD patients, annual influenza vaccination, and COVID-19 vaccination, should be prioritized as cost-effective interventions to reduce infectious complications. Healthcare systems should consider developing SCD-specific vaccination registries and reminder systems to ensure comprehensive coverage, as well as educational initiatives targeting both healthcare providers and patients to emphasize the critical importance of vaccination in this immunocompromised population.

### 4.4. Clinical Outcomes and Prognostic Factors

Despite the complex infectious susceptibility of SCD patients, the clinical outcomes in our cohort were favorable compared to historical reports. The ICU admission rate of 18.8% and in-hospital mortality rate of 3.8% are comparable to or slightly lower than rates reported in studies from Western countries [2,36]. This suggests that adherence to standardized antimicrobial and supportive care protocols can lead to good outcomes even in non-specialized settings.

The finding that patients requiring ICU admission had significantly higher white blood cell counts and lower hemoglobin levels may serve as early predictors of disease severity and the need for intensive monitoring [37]. These laboratory parameters likely reflect the degree of inflammatory response and hemolytic crisis, respectively, both of which are associated with more severe ACS episodes [38].

The 30-day readmission rate of 15% is consistent with reported rates for SCD patients and highlights the chronic nature of this disease and the ongoing risk of recurrent complications [39,40]. The recurrence rate of 20% for ACS emphasizes the importance of long-term preventive strategies, including optimized disease-modifying therapy and infection prevention measures.

### 4.5. Disease-Modifying Therapy and Infection Risk

The low rate of hydroxyurea use at baseline (33.3%) represents a missed opportunity for comprehensive SCD management. Hydroxyurea has been shown to reduce the incidence and severity of ACS episodes, partly through its effects on reducing vaso-occlusive crises and improving overall disease stability [41]. Additionally, hydroxyurea may have immunomodulatory effects that could influence infection susceptibility, although this relationship requires further investigation [42].

Increased utilization of hydroxyurea and other disease-modifying therapies has the potential to reduce both infectious and non-infectious triggers of ACS, making it an essential component of comprehensive SCD care [43]. The integration of hematology specialists in the care of SCD patients could help optimize the use of these therapies and improve overall outcomes [44].

### 4.6. Limitations

This study has several important limitations that must be acknowledged. First, its retrospective design is inherently susceptible to information bias and relies on the accuracy of medical record documentation. The potential for missing or incomplete microbiological data may have led to underestimation of the true infectious burden in ACS episodes.

Second, this was a single-center study conducted at a tertiary care facility, which limits the generalizability of findings to other healthcare settings and patient populations. The patient population at a tertiary center may represent a more severe spectrum of disease compared to community settings.

Third, the sample size of 80 ACS episodes, while providing valuable regional data, limits the statistical power for detecting associations between specific pathogens and clinical outcomes. Larger multicenter studies would be needed to better characterize rare infectious etiologies and their clinical significance.

Finally, the lack of standardized protocols for viral testing and some aspects of microbiological evaluation may have resulted in underdetection of certain pathogens, particularly respiratory viruses that can serve as triggers for ACS. The limited viral testing in our study represents a significant potential confounder, as viral infections are known to be important precipitants of ACS and may have been present in cases where no bacterial pathogen was identified. This limitation highlights the need for comprehensive respiratory viral panel testing as part of the routine workup for ACS in future studies.

### 4.7. Future Directions

Future research should focus on several key areas to advance our understanding of infectious complications in SCD. Prospective multicenter studies with standardized microbiological protocols, including comprehensive viral testing and molecular diagnostic methods, would provide more robust data on the true infectious burden in ACS.

Antimicrobial stewardship research focusing on optimal empirical regimens, duration of therapy, and strategies to minimize resistance development would be valuable for this immunocompromised population. Additionally, studies evaluating the impact of enhanced vaccination programs and their cost-effectiveness in preventing infectious complications are urgently needed.

Biomarker research to identify early predictors of infectious versus non-infectious ACS episodes could guide more targeted antimicrobial therapy and reduce unnecessary antibiotic exposure. The development of rapid diagnostic tools for pathogen identification in ACS could significantly improve clinical decision-making and outcomes.

Finally, the rapidly evolving landscape of SCD therapeutics, including novel anti-sickling agents and gene therapies, presents opportunities to study how these interventions may influence infectious susceptibility and ACS incidence in this population. It is hoped that regulatory agencies will expedite the approval process for breakthrough therapies, particularly given the limited treatment options currently available for SCD patients.

## 5. Conclusions

In this cohort of SCD patients in Eastern Saudi Arabia, ACS demonstrated low rates of specific pathogen identification despite systematic microbiological investigation, supporting the multifactorial infectious and non-infectious etiology of this syndrome. The predominant use of broad-spectrum antimicrobial therapy targeting both typical and atypical bacterial pathogens proved effective in this immunocompromised population, with clinical outcomes comparable to those reported from specialized centers. However, significant gaps in infection prevention strategies were identified, particularly suboptimal vaccination rates against encapsulated bacteria in this functionally asplenic population. These findings emphasize the critical importance of comprehensive vaccination programs and the need for systematic approaches to infection prevention in SCD patients. The study highlights the complex interplay between infectious and non-infectious factors in ACS pathogenesis and underscores the importance of empirical antimicrobial coverage while awaiting microbiological results. Future research should focus on developing more sensitive diagnostic methods for pathogen identification, optimizing antimicrobial stewardship strategies, and implementing comprehensive infection prevention programs to reduce the burden of infectious complications in this vulnerable population.

## Figures and Tables

**Table 1:** Baseline Patient Characteristics of Sickle Cell Disease Patients (N = 60).

Characteristic	Value
Demographics	
Age (years), mean ± SD	24.6 ± 8.3
Male, n (%)	35 (58.3)
SCD Genotype	
Homozygous (HbSS), n (%)	52 (86.7)
Other (HbS-β-thalassemia), n (%)	8 (13.3)
Baseline Therapy	
On Hydroxyurea, n (%)	20 (33.3)
Vaccination Status	
Pneumococcal vaccine, n (%)	30 (50.0)
Annual influenza vaccine, n (%)	18 (30.0)
COVID-19 vaccine (≥1 dose), n (%)	24 (40.0)

**Table 2:** Clinical Presentation of ACS Episodes (N = 80).

Finding	Value
Clinical Setting	
ACS on admission, n (%)	60 (75.0)
ACS developed during hospitalization, n (%)	20 (25.0)
Concurrent Vaso-Occlusive Crisis (VOC), n (%)	72 (90.0)
Presenting Symptoms	
Chest pain, n (%)	64 (80.0)
Shortness of breath, n (%)	60 (75.0)
Fever, n (%)	48 (60.0)
Cough, n (%)	44 (55.0)
Physical Examination	
Tachypnea, n (%)	56 (70.0)
O_2_ saturation < 92% on room air, n (%)	40 (50.0)
Radiological Findings	
Unilateral infiltrate, n (%)	56 (70.0)
Bilateral infiltrates, n (%)	24 (30.0)
Pleural effusion, n (%)	16 (20.0)

**Table 3:** Management Modalities for ACS Episodes (N = 80).

Treatment	Value
Antibiotic Therapy	
Ceftriaxone and Azithromycin, n (%)	76 (95.0)
Escalated antibiotic therapy, n (%)	16 (20.0)
Supportive Care	
Supplemental oxygen, n (%)	44 (55.0)
Non-invasive ventilation, n (%)	5 (6.3)
Other Treatments	
Bronchodilators, n (%)	20 (25.0)

**Table 4:** Microbiological Findings and Clinical Outcomes in ACS Episodes (N = 80).

Finding	Value
Microbiological Findings	
Any identified pathogen, n (%)	8 (10.0)
Positive blood culture, n (%)	5 (6.3)
Positive sputum culture, n (%)	4 (5.0)
Mycoplasma pneumoniae identified, n (%)	3 (3.8)
Clinical Outcomes	
ICU admission, n (%)	15 (18.8)
Mechanical ventilation, n (%)	8 (10.0)
Length of hospital stay (days), median (IQR)	7 (5–11)
In-hospital mortality, n (%)	3 (3.8)
Recurrent ACS, n (%)	12 (20.0)
30-day readmission, n (%)	12 (15.0)

**Table 5:** Comparison of Laboratory Findings Between ICU and Ward Patients.

Characteristic	ICU (n = 15)	Ward (n = 65)	*p*-Value
White blood cell count (×10^9^/L), mean ± SD	18.3 ± 5.1	14.2 ± 3.9	0.004
Hemoglobin (g/dL), mean ± SD	7.2 ± 1.1	8.1 ± 1.2	0.02

## Data Availability

The data presented in this study are available on request from the corresponding author. The data are not publicly available due to privacy and ethical restrictions.
